# Predicting Surgical Site Infections in Spine Surgery: Association of Postoperative Lymphocyte Reduction

**DOI:** 10.3390/diagnostics14232715

**Published:** 2024-12-02

**Authors:** Akiyoshi Miyamoto, Masato Tanaka, Angel Oscar Paz Flores, Dongwoo Yu, Mukul Jain, Christan Heng, Tadashi Komatsubara, Shinya Arataki, Yoshiaki Oda, Kensuke Shinohara, Koji Uotani

**Affiliations:** 1 Department of Orthopedic Surgery, Okayama Rosai Hospital, 1-10-25 Chikkomidorimachi, Minami Ward Okayama, Okayama 702-8055, Japan; akkun@kzd.biglobe.ne.jp (A.M.); angeloscarpaz@gmail.com (A.O.P.F.); ydwnss@yu.ac.kr (D.Y.); drmukuljain@gnail.com (M.J.); heng.christian@gmail.com (C.H.); t.komatsubara1982@gmail.com (T.K.); araoyc@gmail.com (S.A.); 2 Department of Orthopedic Surgery, Okayama University Hospital, Okayama 700-8558, Japan; odaaaaaaamn@yahoo.co.jp (Y.O.); joker1011ks@yahoo.co.jp (K.S.); coji.uo@gmail.com (K.U.)

**Keywords:** surgical site infection, spine surgery, instrumentation, diagnosis, lymphocyte

## Abstract

Objective: Postoperative lymphopenia is reported as an excellent indicator to predict surgical-site infection (SSI) after spine surgery. However, there is still controversy concerning which serological markers can predict spinal SSI. This study aims to evaluate excellent and early indicators for detecting SSI, focusing on spine instrumented surgery. Materials and Methods: This study included 268 patients who underwent spinal instrumented surgery from January 2022 to December 2023 (159 female and 109 male, average 62.9 years). The SSI group included 20 patients, and the non-SSI group comprised 248 patients. Surgical time, intraoperative blood loss, and glycemic levels were measured in both groups. The complete blood cell counts, differential counts, albumin, and C-reactive protein (CRP) levels were measured pre-surgery and postoperative on Days 1, 3, and 7. In comparing the groups, the Mann–Whitney U test analysis was used for continuous variables, while the chi-squared test and Fisher’s exact test were used for dichotomous variables. Results: The incidence of SSI after spinal instrumentation was 7.46% and was relatively higher in scoliosis surgery. The SSI group had significantly longer surgical times (248 min vs. 180 min, *p* = 0.0004) and a higher intraoperative blood loss (772 mL vs. 372 mL, *p* < 0.0001) than the non-SSI group. In the SSI group, the Day 3 (10.5 ± 6.2% vs. 13.8 ± 6.0%, *p* = 0.012) and Day 7 (14.4 ± 4.8% vs. 18.8 ± 7.1%, *p* = 0.012) lymphocyte ratios were lower than the non-SSI group. Albumin levels on Day 1 in the SSI group were lower than in the non-SSI group (2.94 ± 0.30 mg/dL vs. 3.09 ± 0.38 mg/dL, *p* = 0.045). There is no difference in CRP and lymphocyte count between the two groups. Conclusions: SSI patients had lower lymphocyte percentages than non-SSI patients, which was a risk factor for SSI, with constant high inflammation. The Day 3 lymphocyte percentage may predict SSI after spinal instrumented surgery.

## 1. Introduction

Surgical site infection (SSI) is one of the most severe complications following spinal instrumented surgery. This condition often causes significant short- and long-term consequences for patients and sometimes involves considerable socioeconomic burden and revision surgery [1]. Despite many SSI risk studies, there is a lack of definitive conclusive parameters for the early diagnosis of SSI [2]. The aspiration of fluid or tissue biopsy [3], drain tube, and microbiologic culture [4] to confirm the bacteria/fungus is still the gold standard for SSI diagnosis. Imaging modalities such as magnetic resonance imaging (MRI) or enhanced CT are usually helpful, but after instrumentation surgery, the metal artifacts complicate the process of obtaining clear images [5]. Antibiotic stewardship in spine surgery requires the consistent evaluation of antibiotic use for drug selection, dose, and duration to prevent SSI [6]. Most patients with spinal infections diagnosed in early stages can be successfully managed conservatively with antibiotics, bed rest, and spinal braces [7]. However, the SSI treatment of the patient with spinal instrument is very difficult because of biofilm on the metal surface.

Useful inflammatory markers for routine laboratory testing are C-reactive protein (CRP), erythrocyte sedimentation rate (ESR), and white blood cell (WBC) counts. Among these, CRP is superior to others in the assessment of SSI [8]. However, it takes more than ten days to diagnose SSI by CRP, and there is no excellent method to diagnose SSI in the early stage [9]. Recently, several reports have recommended interleukin-6 [10], procalcitonin [11], and TNF-α [12]. For early SSI diagnosis, Iwata et al. reported the usefulness of checking postoperative lymphocytopenia after spinal instrumented surgery [13]. This study aims to evaluate excellent and early indicators for detecting SSI, focusing on spine instrumented surgery.

## 2. Materials and Methods

### 2.1. Study Design

This study was designed as a diagnostic retrospective study based on the pre-and postoperative serological markers and clinical data in spine instrumented surgery. It was approved by the institutional review board of the Okayama Rosai Hospital (Ethics Committee, approval number 435). The necessary informed consents were duly signed and obtained from all the patients involved in this study.

### 2.2. Patients

**Figure 1:**
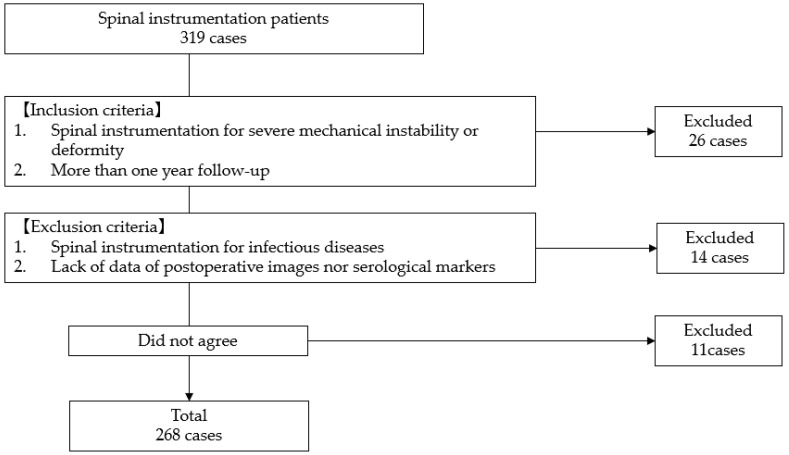
Patient selection.

This is a retrospective cohort analysis of patients who underwent spinal instrumentation surgery for various etiologies at our institute between January 2022 and December 2023. Inclusion criteria: (1) spinal instrumentation for severe mechanical instability or deformity, (2) more than one-year follow-up. Exclusion criteria: (1) spinal instrumentation for infectious diseases such as pyogenic or Tb spondylitis; (2) lack of data of postoperative images or serological markers. This study included two hundred sixty-eight patients (159 females and 109 males, average 62.9 years). These patients were divided into the SSI group and the non-SSI group (Figure 1).

### 2.3. Evaluation

Surgical time, intraoperative blood loss, and glycemic level were checked in both groups. Blood serum samples were collected on the day before the operation or postoperative Days 1, 3, and 7. CRP was measured via immunoturbidimetry (Abbott, Wiesbaden, Germany) with a reference range of <5.0 mg/L. WBC count was determined using the CELL DYN hematology analysis system (Abbott) with a reference range of 4.0–11.0 × 10^3^/µL.

### 2.4. Statistical Evaluation

In comparing the groups, the Mann–Whitney U test analysis was used for continuous variables. In contrast, the chi-squared and Fisher’s exact tests were used for Ordinal scale data in this study and analyzed using the Mann–Whitney U test. In contrast, continuous variables were evaluated using the independent samples *t*-test, a parametric method ideal for comparing the means of two independent groups. All statistical calculations were meticulously performed using GraphPad Prism version 6.0 (GraphPad Software, La Jolla, CA, USA). Statistical significance was set at *p* < 0.05, with this threshold guiding the identification of meaningful differences and associations within the study’s findings.

## 3. Results

### 3.1. Patient Demographics in Both Groups

The average age of the SSI group was 47.9 years, while the average age of the non-SSI group was 64.1 years (*p* = 0.0152). There was a significant difference in age between the two groups. In the SSI group, there were nine males and eleven females. In the non-SSI group, there were 100 males and 148 females. No significant difference in gender distribution was observed between the two groups. The incidence of SSI after spinal instrumentation was 7.46% and was relatively higher for scoliosis surgery (Table 1). The initial surgeries are summarized in Table 2. In the SSI group, there were five posterior cervical fusions, three transforaminal lumbar interbody fusions, and twelve corrective scoliosis surgeries.

### 3.2. Comparison of the Surgical Results Between the Two Groups

**Figure 2:**
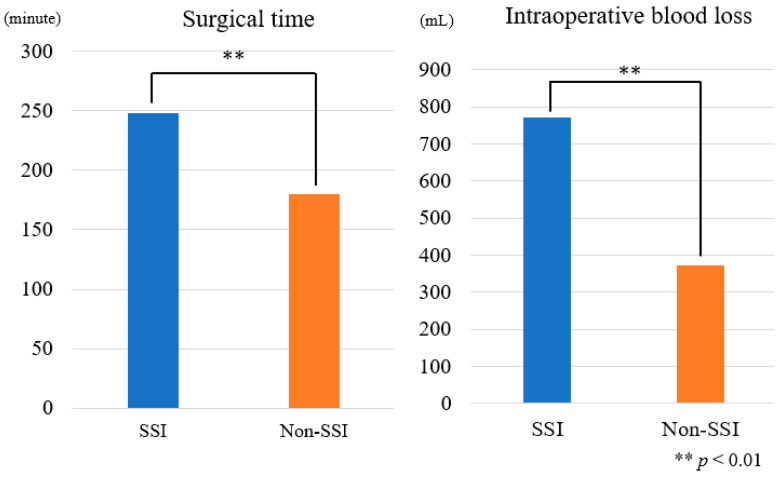
Surgical time and intraoperative blood loss. ** *p* < 0.01.

A comparison of surgical outcomes between the two groups revealed differences in surgical time and average blood loss (ABL). The SSI group had significantly longer surgical times (248 min vs. 180 min, *p* = 0.0004) and higher intraoperative blood loss (772 mL vs. 372 mL, *p* < 0.0001) (Figure 2).

There is no statistical difference between the two groups’ fasting blood sugar and HbA1c levels (Table 3).

The confirmed bacteria and the initial surgeries are summarized in Table 4.

The most common bacteria were MRSA and Serratia.

### 3.3. Comparison of the Serological Markers Between the Two Groups

Both groups’ albumin, CRP, and WBC values are shown in Table 5, Table 6 and Table 7. In the SSI group, Day 1 albumin in the SSI group was significantly lower than that of the non-SSI group (2.94 g/dL vs. 3.09 g/dL, *p* = 0.045). However, there was no difference in WBC in the two groups.

Table 8 shows the lymphocyte percentages of both groups. In the SSI group, the lymphocyte percentages on Day 3 (10.5 ± 6.2% vs. 13.8 ± 6.0%, *p* = 0.012) and Day 7 (14.4 ± 4.8% vs. 18.8 ± 7.1%, *p* = 0.012) was lower than in the non-SSI group. The Day 3 and 7 lymphocyte percentages in the SSI group were significantly lower than those of the non-SSI group (Table 8).

## 4. Discussion

Postoperative surgical site infection (SSI) is among the most dreaded complications in spine surgery. In the retrospective analysis conducted at our institution, the incidence of surgical site infections (SSI) following general instrumented spine surgery was found to be 7.46%, highlighting an area for potential improvement in efforts to reduce infection rates. Despite advancements in surgical techniques and infection control measures, SSIs continue to be a significant concern, with reported infection rates ranging from 0.7% to 17.9% [3]. SSI in spine surgery is particularly concerning due to its potential for severe outcomes, with treatment depending on the severity of the infection. A study published by Elsamadicy et al. corresponding to an analysis of 410,930 patient data showed that SSI is associated with increased morbidity (systemic sepsis, pneumonia, urinary tract infections), prolonged hospital stays (6.5 days vs. 3.0 days), higher 30-day mortality (0.9% vs. 0.4%), and higher healthcare costs [14].

The current study found that patients who developed SSIs had significantly higher intraoperative blood loss (772 mL vs. 372 mL, *p* < 0.0001). Several studies have identified key risk factors for SSI in spine surgery. These include patient characteristics such as advanced age, elevated body mass index (BMI), and underlying comorbidities like diabetes and hypertension [3,15]. Besides patient factors, surgical parameters are critical in developing SSI. The prolonged surgeries increase the patient’s exposure to infectious agents and compromise immune function due to increased blood loss. Intraoperative blood loss and transfusion have been consistently associated with increased risk for SSI [16]. This is consistent with previous findings in the literature, where excessive blood loss during surgery increased the incidence of postoperative infections due to decreased tissue oxygenation and prolonged healing. Excessive blood loss leads to hemodynamic instability that requires transfusion, which further weakens and compromises tissue perfusion and healing.

**Figure 3:**
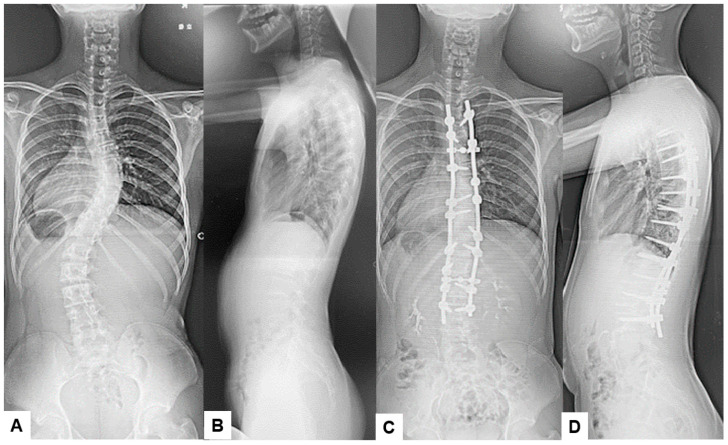
13 M, Adolescent idiopathic scoliosis, Posterior corrective fusion. (**A**) Preoperative spinal posteroanterior radiogram, (**B**) Preoperative spinal lateral radiogram, (**C**) Postoperative spinal posteroanterior radiogram, (**D**) Postoperative spinal lateral radiogram.

**Figure 4:**
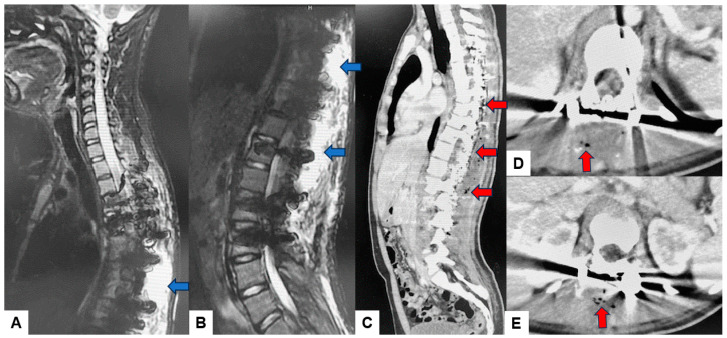
13 M, Adolescent idiopathic scoliosis, MRI and enhanced CT at postoperative Day 12. (**A**) Midsagittal cervicothoracic T2 weighted MR imaging, (**B**) Midsagittal thoracolumbar T2 weighted MR imaging, (**C**) Enhanced midsagittal reconstruction CT, (**D**) Enhanced T8 axial CT, (**E**) Enhanced L1 axial CT. Blue arrows indicate postoperative abscess/effusion. Red arrows revealed gas inside the abscess/effusion.

It is important to note that in our patient sample, no infections were observed in cases involving anterior approaches. This may be attributed to the minimal use of monopolar coagulation and reduced tissue disruption typically associated with anterior approaches. Additionally, our study reinforces the idea that certain types of surgeries, particularly multi-segmental fusions and scoliosis surgeries, may carry a higher risk of SSIs due to their complexity and longer operative times. Figure 3 and Figure 4 represent an example of a case undertaken at our institution for adolescent idiopathic scoliosis, in which the main surgical parameters played a major part in this scenario, and the extensive approach and long operative times resulting in SSI [13,16]. The correlation between extended surgical time and specific pathogens underlines the importance of surgical efficiency and robust perioperative management to mitigate infection risks in spine surgery. In a retrospective meta-analysis undertaken by Zhuo et al. with 22,475 patients, the evidence showed that posterior approaches had double the incidence (5% vs. 2.3%) over anterior approaches, and minimally invasive surgery had a much lower rate of infection (1.5% vs. 3.8%) [17].

Previous reports have emphasized both CE MRIs and CTs can help to identify the underlying microorganisms and differentiate the most frequent subtypes [18]. These image modalities can provide important features to diagnose infectious conditions and to avoid unnecessary biopsy and anti-bacterium treatments. Figure 3 and Figure 4 present the SSI after scoliosis correction surgery. It was a little difficult to use the MRI to evaluate SSI because of metal artifacts. However, enhanced CT revealed gas inside the abscess/effusion which is relatively specific to SSI (Figure 4). 

Notably, one of the emerging findings is the relationship between the duration of surgery and the spectrum of pathogens causing SSI [19]. In our present study, there was a positive association between operating time and the incidence of infection. The SSI group has a significantly higher surgical duration than the non-SSI group (248 min vs. 180 min *p* = 0.0004). This suggests that extended operative times increase the risk of infection and alter the pathogen landscape, potentially leading to more complex, resistant infections. In our sample, the most frequently identified pathogens associated with surgical site infections (SSI) were methicillin-resistant *Staphylococcus aureus* (MRSA) (25%) and *Serratia marcescens* (25%), followed by methicillin-sensitive *Staphylococcus aureus* (12%) and common skin flora. Given that our analysis encompassed a wide variety of procedures, we conclude that the observed dichotomy between the prevalence of Gram-positive and Gram-negative bacteria is attributable to the inclusion of deformity surgeries, which were the most invasive and associated with longer operative durations. A retrospective study by Algarny et al. proved that longer surgeries (more than 200 min) are more likely to expose patients to *Enterococcus faecalis* and *Staphylococcus haemolyticus pathogens*. In comparison, shorter surgeries (less than 120 min) have been associated with pathogens like *Staphylococcus aureus* and *Staphylococcus epidermidis* [19]. Karamian et al. reported in their study with a sample size of 182 SSI patients that gram-negative infections were 10% less likely to require prolonged IV compared with gram-positive bacteria; alongside this finding, mixed infections (46%) did require multiple debridements and extended antibiotic IV therapy [20].

Patient-specific factors such as advanced age, comorbidities like diabetes, elevated body mass index (BMI), and a history of smoking or alcohol consumption increase susceptibility to infections [13,15]. In our study, glycemic control and HbA1c were not correlated with infection. However, this finding should be interpreted cautiously, as our patient sample included various cases, ranging from healthy young patients to elderly, frail individuals with comorbidities. This heterogeneity may have influenced the overall data, masking the association between poor glycemic control and infection risk. A meta-analysis investigated by Zhang et al. reported the risk factors associated with SSI following spinal surgery [16]. A total of 26 studies involving 41,624 patients were included in the analysis, mentioning diabetes (OR = 1.78), hypertension (OR = 1.38), osteoporosis (OR = 2.04), and transfusion (OR = 2.03) as significant risk factors. Other studies have consistently demonstrated that poor glycemic control is a significant risk factor for postoperative complications, including SSIs, such as the one performed by Bakaes et al. who retrospectively analyzed 410 trauma patients undergoing spine surgery and reported that glycemic levels above >200 mg/dL were associated with a statistically significant increase in complications (respiratory problems OR = 2.1, skin/wound complications OR = 2.8, Length of stay OR = 9.6) and poor surgical outcomes [21].

Albumin, a marker of nutritional status, has also been implicated in the development of SSIs. Hypoalbuminemia is associated with impaired wound healing and a weakened immune system. The current study found that albumin levels on postoperative Day 1 were significantly lower in the SSI group (2.94 ± 0.30 mg/dL) compared with the non-SSI group (3.09 ± 0.38 mg/dL). These findings are to be considered but evaluated in the global situation of the patient. There has been a widespread attempt to associate nutritional status and its surgical repercussions; in a study by Quereshi et al., they concluded that screening for this in the preoperative period for patients undergoing major spinal surgery to modify them before the procedure could enhance recovery and prevent complications [22]. The metabolic analysis was focused on total leucocyte count (<1500 cels/mm^3^), albumin (<3.5 g/dL), and transferring (<200 mg/dL) as indicators of poor nutritional or metabolical abnormality status; they reported patients undergoing spinal surgery might benefit from preoperative nutritional optimization [22]. Feng et al. published an observational study of 154 patients and found no significant association between anesthesia methods, albumin levels, or blood glucose levels and the occurrence of SSI [23]. On the contrary, Chaker et al., in their publication of 22,518 patients (15,629 lumbar and 6889 cervical cases), noted that the limit threshold between 3.5 and 4.0 g/dL albumin was associated with an increase in readmission and length of stay. Specifically, the cervical patients with albumin between 3.5 g/dL and 3.7 g/dL were at higher risk of SSI (OR = 1.82) and readmission [24].

Inflammatory markers, such as C-reactive protein (CRP) and lymphocyte counts, have become early diagnostic tools for detecting SSIs. Elevated CRP levels and lymphopenia observed in the early postoperative period are valuable indicators of infection [13,25]. However, CRP was not a good indicator for detecting early SSI in this study. This issue is still controversial because another study has also confirmed that CRP is not a good indicator for early SSI [26]. The current study supports that lymphopenia is an excellent indicator for detecting early SSI. In the SSI group, the lymphocyte percent on Day 3 (10.5 ± 6.2% vs. 13.8 ± 6.0%, *p* = 0.012) and Day 7 (14.4 ± 4.8% vs. 18.8 ± 7.1%, *p* = 0.012) was lower than in the non-SSI group. This is consistent with findings that postoperative lymphopenia reflects immune suppression, heightening the risk of bacterial invasion [13,15,25]. Ji et al. highlighted that a lymphocyte count of less than 1.16 × 10⁹/L at three days postoperatively offers substantial diagnostic value for infections, even outperforming CRP in some cases [15]. Iwata et al. published two studies regarding serological markers; the first one involved 302 patients, out of which, 12 developed deep SSIs and were screened with key markers that measured temporal changes regarding C-reactive protein (CRP), WBC count, neutrophil count, and lymphocyte count [25]. The combination of these markers showed moderate sensitivity (50%) and high specificity (81%) [25]. In the other study, five patients that developed an SSI out of a sample size of 85 were analyzed in a case-control scenario, in which six laboratory markers were included; from those, CRP over 10 mg/dL at day four and Lymphocyte counter 1000/μL at day 4 were the most relevant [13]. The CRP at post-surgery Day 4 was the most specific (97.5%), and the lymphocyte count at Day 4 post-surgery was the most sensible (80%) [13].

There have also been attempts to publish a scoring system to predict surgical site infection in the spine. Imabayashi et al. proposed a scoring system based on a combination of four markers: neutrophil count, NLR on day 7 lymphocyte count ratio, and CRP ratio, giving each value a point if the value is above the cutoff [26]. A score of 3 or more points (based on these four markers) was highly predictive of SSI, with a sensitivity of 89% and specificity of 92%. When combined with the early serological markers, such scoring models could significantly improve clinical decision-making, enabling timely intervention and reducing the risk of postoperative complications.

### Study Limitations

There were several limitations to this study. As a retrospective study, there is an inherent risk of selection bias and incomplete data collection. This study included only Japanese patients and was conducted at a single center, which may limit the applicability of the results to other clinical settings. The number in the SSI group was larger than in the non-SSI group, which might bias statistical evaluation. Additionally, the heterogeneity of the patient population, including healthy young individuals and elderly patients with comorbidities, could have influenced the outcomes. This diversity makes isolating the impact of specific risk factors, such as glycemic control, on developing SSI challenging.

## 5. Conclusions

Our findings reaffirm that longer surgeries and greater blood loss are closely associated with a higher incidence of SSI, while the type of surgery, particularly multi-segmental fusions and scoliosis procedures, further elevates the risk.

Regarding early diagnosis of SSI, lymphopenia, particularly on postoperative Days 3 and 7, proved a sensitive marker of infection, underscoring the importance of immune monitoring in postoperative care. Additionally, the impact of hypoalbuminemia on infection risk emphasizes the necessity of addressing nutritional status in perioperative management.

## Figures and Tables

**Table 1:** Patient demographics in both groups.

	SSI	Non-SSI	*p* Value
Male	9	100	1
Female	11	148	
Total	20	248	
Age	47.9 ± 26.3	64.1 ± 20.9	0.0152 *

* *p* < 0.05.

**Table 2:** Initial surgery.

	SSI (*n* = 20)	Non-SSI (*n* = 248)
Cervical Anterior	0	21
Cervical Posterior	5	52
Thoracic Anterior	0	3
Thoracic Posterior	0	13
Lumbar Anterior	0	8
Lumbar Posterior	3	42
Lumbar Anterior and Posterior	0	72
Scoliosis	12	37

**Table 3:** The fasting blood sugar and HbA1c of both groups.

	SSI (*n* = 20)	Non-SSI (*n* = 248)	*p*-Value
Fasting blood sugar	1108.6 ± 25.5 g/dL	114.1 ± 38.0	0.842
HbA1c	5.77 ± 0.49	5.99 ± 0.78	0.220

**Table 4:** The confirmed bacteria and the initial surgeries.

Bacteria			
MRSA	5	Staphylococcus epidermidis	1
Serratia marcescent	5	Enterococcus faecalis	1
MSSA	2	Propionibacterium acnes	1
Staphylococcus caprae	1	Not detectable	4

**Table 5:** The albumin values of both groups.

	SSI (*n* = 20)	Non-SSI (*n* = 248)	*p*-Value
Preoperation	4.41 ± 0.39 g/dL	4.11 ± 0.51	0.005 **
Day 1	2.94 ± 0.30	3.09 ± 0.38	0.045 *
Day 3	2.86 ± 0.30	2.95 ± 0.37	0.234
Day 7	3.18 ± 0.46	3.29 ± 0.42	0.365

* *p* < 0.05, ** *p* < 0.01.

**Table 6:** The CRP values of both groups.

	SSI (*n* = 20)	Non-SSI (*n* = 248)	*p*-Value
Preoperation	0.139 ± 0.13	0.578 ± 1.48	0.858
Day 1	2.37 ± 1.80	3.93 ± 2.87	0.0027 **
Day 3	11.3 ± 8.12	10.2 ± 5.67	0.809
Day 7	3.61 ± 4.14	2.24 ± 2.31	0.430

** *p* < 0.01.

**Table 7:** The WBC values of both groups.

	SSI (*n* = 20)	Non-SSI (*n* = 248)	*p*-Value
Preoperation	6657 ± 1831	6641 ± 1985	0.896
Day 1	8385 ± 2139	8849 ± 2330	0.538
Day 3	9453 ± 3030	8237 ± 2091	0.110
Day 7	7363 ± 2204	6754 ± 2020	0.161

**Table 8:** The lymphocyte percentages of both groups.

	SSI (*n* = 20)	Non-SSI (*n* = 248)	*p*-Value
Preoperation	26.1 ± 10.0	27.1 ± 9.9	0.753
Day 1	10.9 ± 4.6	11.9 ± 5.5	0.496
Day 3	10.5 ± 6.2	13.8 ± 6.0	0.0119 *
Day 7	14.4 ± 4.8	18.8 ± 7.1	0.0122 *

* *p* < 0.05.

## Data Availability

Dataset available on request from the authors. The raw data supporting the conclusions of this article will be made available by the authors on request.
